# Effects of Aflatoxin on Some Haematological Parameters and Protective Effectiveness of Esterified Glucomannan in Merino Rams

**DOI:** 10.1100/2012/342468

**Published:** 2012-04-26

**Authors:** Nurcan Dönmez, H. H. Dönmez, E. Keskin, İ. Kısadere

**Affiliations:** ^1^Department of Physiology, Faculty of Veterinary Medicine, University of Kyrgyzstan-Turkey Manas, Bishkek 720044, Kyrgyzstan; ^2^Department of Histology and Embryology, Faculty of Veterinary Medicine, University of Kyrgyzstan-Turkey Manas, Bishkek 720044, Kyrgyzstan; ^3^Department of Physiology, Faculty of Veterinary Medicine, Selçuk University, 42075 Konya, Turkey

## Abstract

The objective of the present study was to evaluate the toxic effects of aflatoxin on some hematological parameters and to determine the preventive effectiveness of added glucomannan. In the study, 32 Merino rams were used, and the rams were separated equally to four groups as control (C), glucomannan (G), glucomannan + aflatoxin (AG), and aflatoxin (A). Erythrocyte, leukocyte count, hemoglobin, and hematocrit levels were decreased in A group compared with the other groups, and there was a reduction in similar parameters in AG group compared to control values. On the other hand, these parameters were tended to increase in AG group compared to A group values. Aflatoxicosis caused the lymphocytopenia and monocytopenia but increased percentage of neutrophil counts. In conclusion, the results determined in the study might be important to demonstrate the effects of aflatoxicosis and glucomannan on some haematological parameters before the clinical symptoms appear.

## 1. Introduction

One of the most important problems that occurs as a result of unconditioned storage of food and foodstuff is toxications caused by mycotoxins [1]. Mycotoxins are toxic metabolic by-products, which are produced by fungi. Naturally fungi on crops produce mycotoxins in the fields, during harvest and in storage. Contamination of food or feedstuffs and their consumption can result in mycotoxicosis. In these mycotoxins, aflatoxins are the mostly seen and aflatoxin B1 is the most harmful one [2, 3]. Synthesis of aflatoxins in feeds are increased at temperatures above 27°C (80 F), humidity levels greater than 62%, and moisture levels in the feed above 14% [4]. Consumption of aflatoxin-contaminated food by human and animals causes important health problems together with important economical losses. All animal species are susceptible to aflatoxicosis, but outbreaks occur mostly in pigs, sheep, and cattle. Beef and dairy cattle are more susceptible to aflatoxicosis than sheep or horses [5]. Aflatoxicosis causes several defects in organs and tissues, decrease in growth rate, increase in death rate, immunosuppression, anemia, and increase in coagulation time and deteriorates lipid, carbohydrate, and protein metabolism [1, 6].

As a result of toxic effect of aflatoxin, biochemical and hematological parameters have been reported to be changed importantly. In chronic and subclinical aflatoxicosis case, changes in biochemical and hematological parameters occur before clinical symptoms develop [7, 8]. Significant changes in serum biochemical and hematological parameters are seen in aflatoxicosis cases, and these can assist in the diagnosis of toxications [9, 10].

Removing AF from contaminated food and foodstuffs remains a major problem, and there is a great demand for effective decontamination technology. Decontamination procedures have focused on degrading, destroying, inactivating, or removing AF by physical, chemical, or biological methods [10, 11]. Use of adsorbents (silicates) or chemicals (ammonia, sodium bisulfate) or biological agents (Saccharomyces cerevisiae) in animal feeds has been shown to minimize the untoward effect of aflatoxin. Esterified glucomannan (EG) showed very high binding ability (80–97%) with AF [9, 12]. The studies performed with EG at different concentrations of AF showed that EG partially or completely reversed the effect of AF on performance, biochemistry, hematology, and immune response [6, 7, 13].

In the present study, the effects of aflatoxin on some hematological parameters were determined in ram fed feed containing aflatoxin. Furthermore, the effect of adding glucomannan, which is a known aflatoxin binder, on the negative effects of aflatoxin was studied, as well.

## 2. Materials and Methods

In the present study, 32 healthy Merino rams (3 years old) were used. The rams were divided into 4 equal groups. Body weight of groups were close to each other and given in Table 1 before and after experiment.

During the experiment (12 weeks), the rams received clean water continuously and were fed *ad libitum* as follows.

Group 1 (C): fed with the normal diet for rams.
Group 2 (A): fed with the food containing 250 *μ*g/day aflatoxin.
Group 3 (G): fed with the food containing 2 g/day glucomannan.
Group 4 (AG): fed with the food containing 250 *μ*g/day aflatoxin + 2 g/day glucomannan.

Aflatoxin and glucomannan were given to rams by mixing to 100 gr diet before morning feeding. When the rams finished the mixed diet, the rest feed was given.

At the end of the experiment, blood samples were collected into tubes with added anticoagulant. The red blood cell (RBC), white blood cell (WBC), hematocrit values, haemoglobin levels, and differential leukocyte counts were determined. The red blood cell (RBC) and white blood cell (WBC) were determined by haematocytometer; haemoglobin amounts were determined by commercial kit (Biosystem), and differential leukocyte counts were determined in blood smears [14].

Statistical differences among the groups were tested by analysis of variance (ANOVA) which is followed by Duncan's test using SPSS for windows version 16.0 [15]. Significants were considered as *P* < 0.05.

## 3. Results and Discussion

Results obtained from all groups were given in Tables 2 and 3.

Chronic aflatoxicosis may be diagnosed by determining serum biochemical and hematological alterations before clinical symptoms become apparent [11]. Even small amounts of AF are dangerous for animal health because of detrimental effect on some biochemical and hematological values [16].

In the present study, erythrocyte count, leukocyte count, hemoglobin, and hematocrit levels were decreased in A group compared with the other groups. But this reduction was significantly (*P* < 0.05) important in RBC, WBC, and hemoglobin in A group compared with control, and there was a reduction in similar parameters in AG group compared to control values. On the other hand, these parameters were tended to increase in AG group compared to A group values (Table 2).

Keçeci et al. [16] have reported that the hematocrit and hemoglobin levels, MCH, and thrombocyte counts were depressed and increased the sedimentation rate by AF in broilers, and Oğuz et al. [11] reported that decreased were hematocrit and hemoglobin levels, MCV, erythrocyte, thrombocyte, and lymphocyte counts. Furthermore, in another study, it has been reported that AF caused decrease in hematocrit and hemoglobin levels, MCV, erythrocyte, thrombocyte, and lymphocyte counts, and glucomannan (1 g/kg) depressed these negative effects of AF in broilers [9]. Our findings supported statement of above studies, and Yousef et al. [17] and Abdel-Wahhab et al. [2] have reported similar changes to our data.

Abdel-Wahhab et al. [2] have reported that decrease in hemoglobin concentration and total red blood cell counts resulting in normocytic normochromic anaemia in aflatoxin application alone. This decrease in the hematological parameters may be due to many factors such as inhibition of protein synthesis as evidenced by lower serum albumin [18], decrease of the total iron binding capacity [19], and the hemopoietic cellular defects of AF [2, 20].

Furthermore, broiler chicks given 2,5 mg AF/kg diet have shown not only decreased hemoglobin, and hematocrit values, thrombocyte counts, percentage of lymphocyte and basophil counts but also increased percentage of heterophils [21]. Again, the researchers reported that aflatoxicosis caused the lymphocytopenia and monocytopenia but increased the WBC and percentage of heterophil counts [2, 9, 11, 22]. This increase in WBC and percentage of heterophil counts suggest that the toxin elicited an inflammatory response. However, the decreases in the other percentage of leukocyte types may be related with relative reduction [1, 16]. There was significant increase (*P* < 0.05) in percentage of neutrophil and decrease in percentage of lymphocyte and monocyte counts in A group compared to control in this study (Table 3).

The using of agents that act as antidotes or antagonize the effects of toxic substances such as AF has therapeutic and economic importance. The major advantages of these absorbents include expense, safety, and easy administration through addition to foods [11], and glucomannan was selected for use in the present study. We considered that toxin absorption from the gastrointestinal tract can be prevented by formation of complex with AF. In the study, there were no significant differences in hematological parameters compared to control group (Tables 2 and 3).

In conclusion, AG group's levels being between G and A groups have showed that glucomannan at this level has moderate counteracts to this aflatoxin dosage in respect of these parameters. Thus, it needs the other studies in different aflatoxin and glucomannan levels. As a result, findings presented in this study contribute to the overall literature, and also chronic intoxications can be diagnosed before clinical signs occurrence. The effectiveness of glucomannan at this dose is believed to be important.

## Figures and Tables

**Table 1:** Body weight of groups before and after experiment (*n* = 8).

Groups	Before experiment	After experiment
C	57.65 ± 0.94	80.93 ± 1.51
G	57.70 ± 1.18	81.96 ± 1.86
A	57.90 ± 0.87	75.69 ± 3.46
AG	57.85 ± 1.32	80.65 ± 1.17

**Table 2:** Effects of AF and glucomannan on some haematological parameters (*n* = 8, X ± SX).

Parameters	C	G	A	AG
Erythrocyte (×10^6^/mm^3^)	11.32 ± 2.86^a^	11.10 ± 3.15^a^	9.42 ± 3.60^b^	10.90 ± 2.87^a^
Leukocyte (×10^3^/mm^3^)	10.87 ± 6.01^a^	10.40 ± 6.42^a^	8.01 ± 7.09^b^	9.38 ± 4.91^ab^
Haematocrit (%)	29.62 ± 1.33	29.50 ± 1.73	25.50 ± 0.86	28.25 ± 1.68
Haemoglobin (gr/dL)	11.87 ± 0.21^a^	11.58 ± 0.54^ab^	9.58 ± 0.40^c^	10.62 ± 0.25^bc^

^a,b,c^ *P* < 0.05.

**Table 3:** Effects of AF and glucomannan on differential leukocyte counts (*n* = 8, X ± SX).

	C	G	A	AG
Neutrophile	33.0 ± 1.47^b^	31.63 ± 1.03^b^	40.63 ± 2.21^a^	34.75 ± 2.16^b^
Lymphocyte	63.13 ± 1.20^a^	64.25 ± 0.99^a^	57.50 ± 2.18^b^	62.12 ± 2.20^ab^
Monocyte	2.12 ± 0.35^a^	1.87 ± 0.58^a^	0.63 ± 0.26^b^	1.75 ± 0.36^ab^
Eosinophil	1.25 ± 0.31	1.75 ± 0.36	0.87 ± 0.29	1.00 ± 0.26
Basophile	0.50 ± 0.18	0.50 ± 0.18	0.37 ± 0.18	0.38 ± 0.18

^a,b^ *P* < 0.05.
